# Left Atrial Anatomy Relevant to Catheter Ablation

**DOI:** 10.1155/2014/289720

**Published:** 2014-06-24

**Authors:** Damián Sánchez-Quintana, José Ramón López-Mínguez, Yolanda Macías, José Angel Cabrera, Farhood Saremi

**Affiliations:** ^1^Department of Anatomy and Cell Biology, Faculty of Medicine, University of Extremadura, 06006 Badajoz, Spain; ^2^Department of Cardiology, Hospital Infanta Cristina, 06080 Badajoz, Spain; ^3^Department of Cardiology, Hospital Quirón, European University of Madrid, 28223 Madrid, Spain; ^4^Department of Radiology, University of Southern California, Los Angeles, CA 90089, USA

## Abstract

The rapid development of interventional procedures for the treatment of arrhythmias in humans, especially the use of catheter ablation techniques, has renewed interest in cardiac anatomy. Although the substrates of atrial fibrillation (AF), its initiation and maintenance, remain to be fully elucidated, catheter ablation in the left atrium (LA) has become a common therapeutic option for patients with this arrhythmia. Using ablation catheters, various isolation lines and focal targets are created, the majority of which are based on gross anatomical, electroanatomical, and myoarchitectual patterns of the left atrial wall. Our aim was therefore to review the gross morphological and architectural features of the LA and their relations to extracardiac structures. The latter have also become relevant because extracardiac complications of AF ablation can occur, due to injuries to the phrenic and vagal plexus nerves, adjacent coronary arteries, or the esophageal wall causing devastating consequences.

## 1. Introduction

There continues to be a lack of understanding of the pathogenesis of AF. Current evidence suggests that the pathogenesis of AF is multifactorial, because this arrhythmia may not only accompany a variety of pathological conditions, but also occur in a heart with no known structural abnormality, a condition known as “lone AF” [1]. Recent decades have seen rapid developments in arrhythmia treatment, especially with the use of catheter ablation approaches. These techniques in patients with AF have evolved from an initial simple approach focused on the pulmonary veins (PVs) and their junctions with the LA, to a more extensive intervention mainly, but not exclusively, targeting the left atrial myocardium [2]. Because the LA is the main target of catheter ablation in patients with AF, in this review we examined the gross morphological and architectural features of this chamber and discussed the importance of its relations to neighboring extracardiac structures.

## 2. Components of the Left Atrium and Its Walls

From a gross anatomical viewpoint, the LA has four components [3]: (1) a venous part that receives the PVs; (2) a vestibule that conducts to the mitral valve; (3) the left atrial appendage (LAA); and (4) the so-called interatrial septum (IAS). The body of LA is interposed between the vestibular and pulmonary venous components, with the PVs entering at the four corners of the venous part, enclosing a prominent atrial dome. The mean left atrial anteroposterior diameter is 38.4 ± 4.9 mm in normal subjects and will be increased with atrial fibrillation (range 44–74 mm). LA volume is larger in persistent AF (159.7 ± 57 mL) compared with paroxysmal AF (129.6 ± 44 mL) [4–6]. With atrial enlargement, the relative position distance of the esophagus to the left pulmonary veins may be increased but this is variable. The relative position of the left phrenic nerve to the left atrial appendage may change with LAA enlargement.

**Figure 1:**
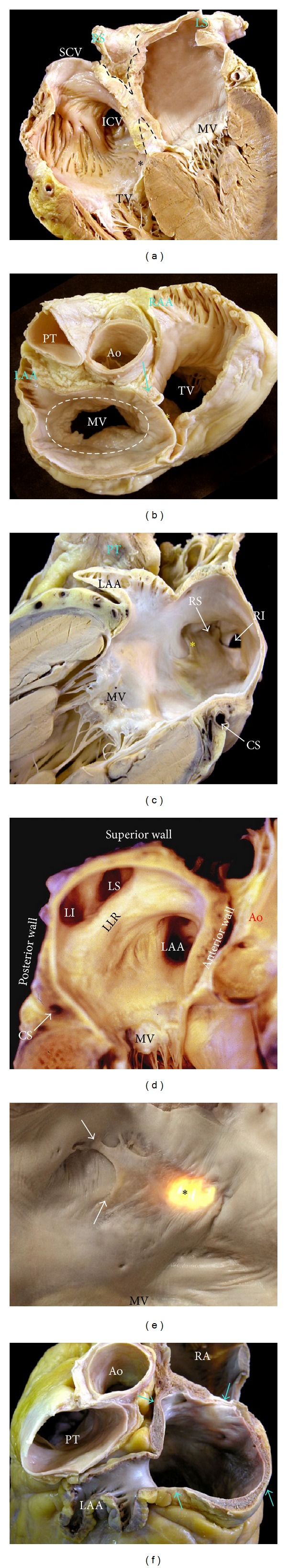
(a) Four-chamber section through the heart showing the offset arrangement of the mitral valve and tricuspid valve which produces the so-called muscular atrioventricular septum (∗) and the deep infolding of the atrial wall superior and inferior to the floor of the oval fossa (dotted lines). (b) Short axis section across the atrial chamber to show the thin flap valve (∗) and the muscular rim of the oval fossa (arrow). Note the atrioventricular valves, the vestibule of the left atrium (dotted line), and the different shape and size of the atrial appendages. (c) and (d) Longitudinal sections through the pulmonary venous component showing the orifices of the right and left PVs and the ostium of the left atrial appendage; the flap valve of the oval fossa overlaps (∗) the rim to form the septal aspect of the left atrium. (e) A magnification of the left aspect of the interatrial septum. Note that, apart from a small crescent-like edge (arrows), the left atrial side of the septum can be seen by transillumination of the oval fossa (∗) in the right side. In the case of patent foramen oval, the LA can be accessed from the right atrium (RA) through a crevice (∗) that is the last part of the valve to be sealed to the rim. (f) Short axis section across the left atrial chamber. Note the nonuniform thickness (arrows) of the left atrial wall. MV = mitral valve, TV = tricuspid valve, SCV = superior cava vein, ICV = inferior cava vein, RAA = right atrial appendage, LAA = left atrial appendage, PT = pulmonary trunk, Ao = aorta, LI = left inferior pulmonary vein, LLR = left lateral ridge, LS = left superior pulmonary vein, RI = right inferior pulmonary vein, RS = right superior pulmonary vein, and CS = coronary sinus.

An anatomic septum in a heart is like a wall that separates adjacent chambers so that its removal would enable us to enter from one chamber to the other without exiting the heart. Thus, the true IAS wall is confined to the flap valve of the oval fossa. The flap valve is hinged from the muscular rim of IAS that, deriving from the septum secundum, is seen from the right atrial aspect of the interatrial wall [3]. At its anteroinferior aspect, the muscular rim separates the oval fossa from the coronary sinus and the vestibule of the tricuspid valve (Figure 1). On the left atrial side of septum, there is no visible rim and the flap valve overlaps the oval rim quite considerably and two horns mark the usual site of fusion with the rim (Figure 1(c)). Therefore, we would like to emphasize that the true IAS is the oval fossa, a depression in the right atrial side of septum traditionally considered to be the IAS. The rest of muscular rim of the IAS is formed by the invagination of the right and left atrial myocardia that are separated by vascularized fibrofatty tissues of the extracardiac fat. This is why we prefer to use the term “interatrial groove” rather than muscular IAS, a concept that is very important during percutaneous interventions because transseptal punctures through the IAS to access the LA should be delimited to the boundary of the oval fossa. Thus, an inadvertent puncture throughout the interatrial groove (muscular IAS) may result in hemopericardium especially in a highly anticoagulated patient because blood will dissect the vascularized fibrofatty tissue that is sandwiched between the right and left atrial walls at this level [7]. The location and size of the oval fossa varies from case to case, as does the profile or prominence of the muscular rim [8]. The interatrial septum has a left ward angle of 45–60° relative to the horizontal plane. This orientation will be different and becomes more horizontal with right pneumonectomy, aortic aneurysm, or a large pleural effusion. Furthermore, abnormalities of the thorax or of the cardiovascular system such as kyphoscoliosis, marked left ventricular hypertrophy, or an enlarged aorta may result in displacement of the oval fossa [9].

The major part of the endocardial LA including the septal wall and interatrial groove component is relatively smooth. The left aspect of the interatrial groove, apart from a small crescent-like edge (Figure 1(e)), is almost indistinguishable from the parietal atrial wall. The smoothest parts are the superior and posterior walls, which make up the pulmonary venous component and the vestibule surrounding the mitral orifice. Behind the posterior wall of the vestibular component of the LA is the anterior wall of the coronary sinus [7] (Figures 1(c) and 1(d)).

The walls of LA are nonuniform in thickness (Figure 1(f)) and in general appear thicker than the right atrium. The walls can be described as being anterior, superior, left lateral, septal, and posterior. The anterior wall is located behind the ascending aorta and the transverse pericardial sinus. The anterior wall thickness measures 3.3 ± 1.2 mm in unselected postmortem hearts [10]. Part of the anterior wall immediately inferior to the Bachmann bundle and posterior to the aorta can be very thin (1-2 mm). The roof or the superior wall is in close proximity to the right pulmonary artery with a mean thickness of 4.5 ± 0.6 mm. The lateral wall thickness is 3.9 ± 0.7 mm. In normal hearts, the anteroinferior rim of the IAS measures 5.5 ± 2.3 mm and the flap valve measures 1.5 ± 0.6 mm [10]. The posterior wall thickness is greatest inferiorly, at 6.5 ± 2.5 mm, when measured immediately superior to the coronary sinus and between 6 and 15 mm from the mitral annulus. By contrast, it is thinnest, at 2.2 ± 0.3 mm, at the right or left venoatrial junctions [11]. In some samples of histological sections obtained at the PV and posterior atrial wall, small areas of discontinuities are seen in the myocardial layer replaced with fibrous tissue.

## 3. The Myoarchitecture of the Left Atrium

**Figure 2:**
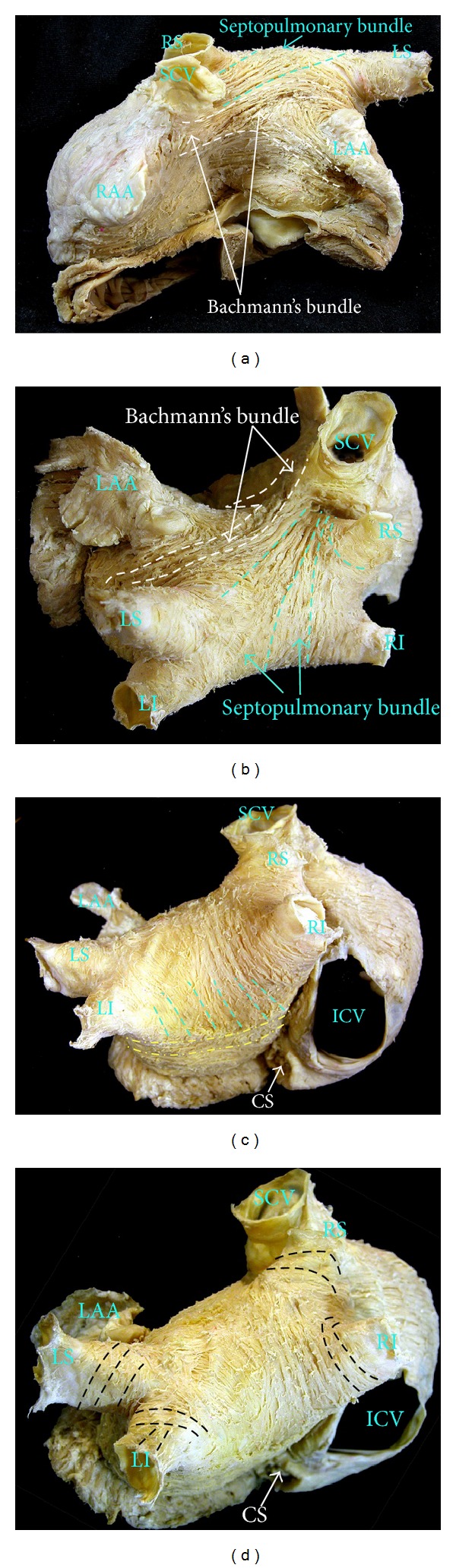
Serial dissections to display the atrial myoarchitecture in a normal human heart. (a) and (b) Anterior and superior views to show the Bachmann bundle crossing the anterior interatrial groove and branching toward the left atrial appendage and note also the longitudinal fibers of the septopulmonary bundle, which arises from the interatrial groove underneath Bachmann's bundle, fanning out to line the pulmonary veins and to pass longitudinally over the dome and in the posterior wall of the left atrium. (c) On the posterior wall, the septopulmonary bundle often crossing the circumferential myocytes coming from the lateral wall which show an abrupt change of fiber orientation in the posterior wall of the left atrium. (d) Note the highly variable anatomic orientation of the myocardial fibers making up the sleeves. The myocytes sleeves are mainly composed of circularly orientated bundles; oblique and longitudinally oriented fibers are also common. SCV = superior cava vein, ICV = inferior cava vein, RAA = right atrial appendage, LAA = left atrial appendage, LI = left inferior pulmonary vein, LLR = left lateral ridge, LS = left superior pulmonary vein, RI = right inferior pulmonary vein, RS = right superior pulmonary vein, and CS = coronary sinus.

Detailed dissections of the subendocardial and subepicardial myofibers along the entire thickness of the LA walls have shown a complex architecture of overlapping bands of aligned myocardial bundles [12, 13] (Figure 2). The term “fibers” describes the macroscopic appearance of strands of cardiomyocytes. These fibers are circumferential when they run parallel to the mitral annulus and longitudinal when they are approximately perpendicular to the mitral orifice.

Although there are some individual variations, our epicardial dissections of the LA have shown a distinctive pattern of arrangement of the myocardial fibers [12]. On the subepicardial aspect of LA, the fibers in the anterior wall consisted of a main bundle that ran parallel to the atrioventricular groove. This was the continuation of the interatrial bundle (Bachmann bundle) [12, 13], which could be traced rightward to the junction between the right atrium and the superior caval vein (Figures 2(a) and 2(b)). In the LA, the interatrial bundle was joined inferiorly at the septal raphe (the portion that is buried in the atrial septum) by fibers arising from the anterior rim of the oval fossa. Superiorly, it blended with a broad band of circumferential fibers that arose from the anterosuperior part of the septal raphe to sweep leftward into the lateral wall. Reinforced superficially by the interatrial bundle, these circumferential fibers passed to either side of the neck of the atrial appendage to encircle the appendage and reunited as a broad circumferential band around the inferior part of the posterior wall to enter the posterior septal raphe.

The epicardial fibers of the superior wall are composed of longitudinal or oblique fibers, (named by Papez as the “septopulmonary bundle” in 1920) [14] (Figures 2(a), 2(b), and 2(c)) that arise from the anterosuperior septal raphe, beneath the circumferential fibers of the Bachmann bundle. As they ascend the roof, they fan out to pass in front of, between, and behind the right and left PVs and the myocardial sleeves that surround the venous orifices. On the posterior wall, the septopulmonary bundle often bifurcates to become two oblique branches. The leftward branch fused with, and was indistinguishable from, the circumferential fibers of the anterior and lateral walls, whereas the rightward branch turned into the posterior septal raphe.

On the subendocardial aspect of LA, most specimens showed a common pattern of general architecture. The dominant fibers in the anterior wall were those originating from a bundle described by Papez as the septoatrial bundle [14]. The fibers of this bundle ascended obliquely from the anterior interatrial raphe and combined with longitudinal fibers arising from the vestibule. They passed the posterior aspect of the LA between the left and right pulmonary veins, blending with longitudinal or oblique fibers of the septopulmonary bundle from the subepicardial layer. The septoatrial bundle also passed leftward, superior, and inferior to the mouth of the LAA to reach the lateral and posterior walls. Some of these fibers encircled the mouth of the LAA and continued into the pectinate muscles within the appendage.

Atrial fibrillation is the most common sustained cardiac arrhythmia and is characterized by uncoordinated contraction of the atrium. It is still unclear whether the initiation and maintenance of human AF depends on automatic focal or reentrant mechanisms. Recent reports have shown the contribution of different atrial regions on the fibrillatory process and to the maintenance of AF, emphasizing the role of structural discontinuities and heterogeneous fiber orientation favoring anatomic reentry or anchoring rotors [15, 16]. The posterior wall of the LA, for example, seems to play an important role in maintaining AF. Morillo et al. [17] reported in a canine model of AF that cryoablation at sites of short cycle length activity in the posterior LA resulted in the interruption of this arrhythmia. Observations from the laboratory of Jalife and coworkers [18] demonstrated in the isolated sheep heart the presence of a small number of stable ongoing circuits generating high frequency waves and providing a base to generate fibrillatory conduction. Data derived from high resolution optical mapping and histological sections in this animal model also showed that the focal sources correspond to single or a small number of reentrant rotors discharging at a high frequency and that these are localized in the PV orifices or at the contiguous posterior left atrial region [19]. Postmortem examination in human specimens showed in most hearts an abrupt change of subendocardial fiber orientation (circumferential, oblique, and longitudinal) in the posterior wall of the LA (Figure 2(c)) at the venoatrial junctions. In these areas, the subendocardial fibers are usually loop-like extensions from the longitudinal fibers encircling the venoatrial junctions [12, 13]. The finding of changes in myoarchitecture transmurally is also relevant. The most obvious broad band or linear anatomic barrier of longitudinal and oblique fibers was formed by the septopulmonary bundle that also marked a change in LA wall thickness. Left atrial endocardial activation was mapped in 19 patients with a percutaneous noncontact mapping system during episodes of focal initiation of AF [20]. In this study, Markides et al. [20] observed that the pattern of LA activation was predominantly determined by a principal line of conduction block. It appears to be related to the linear anatomic barrier identified by the examination of fiber orientation at the level of the septopulmonary bundle.

## 4. Pulmonary Veins and the Atrial Fibrillation Ablation

Although different mechanisms of AF exist, it is well established that the myocardial sleeves of the PVs, especially the superior veins, are crucial sources of triggers that initiate AF [2]. Cardiac ablation is performed in symptomatic AF. Furthermore, patients with larger LA size and longer AF duration typically experience a higher incidence of AF recurrence [2]. Previously, the most common ablation strategy was electrical isolation of the PVs by creating circumferential ablation lines around the individual or bilateral PV ostia [21]. However, the focus of ablation strategies shifted from the PV ostium to the atrial tissue located in the venoatrial junctions due to the fact that many non-PV trigger points for AF are located in the venoatrial junctions rather than the PV and that radiofrequency catheter ablation (RFCA) techniques may cause PV stenosis [22].

**Figure 3:**
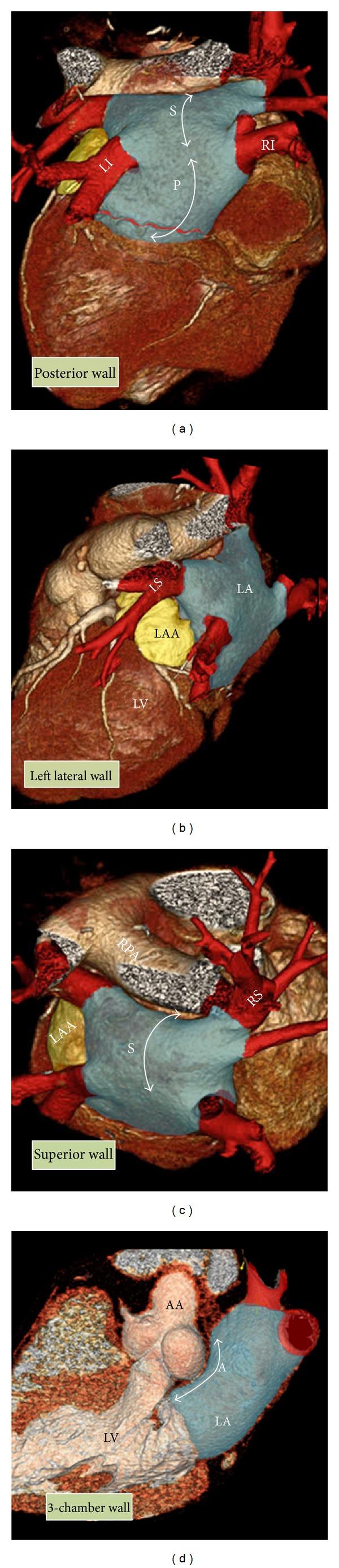
Anatomic relationship of the left atrium (LA). Posterior, right lateral, superior, and three-chamber view of volume rendered CT angiographies are shown. The LA (in blue) is located superiorly and posteriorly to the heart. The walls of LA include the anterior, superior, left lateral, septal, and posterior. Its superior (S) and posterior (P) walls are shown by double-headed arrows. Note close relation of the anterior (A) wall to the ascending aorta (AA). The left atrial appendage (LAA) is shown in yellow and the pulmonary veins are shown in red. The four pulmonary veins include LS = left superior, LI = left inferior, RS = right superior, RI = right inferior, LV = left ventricle.

**Figure 4:**
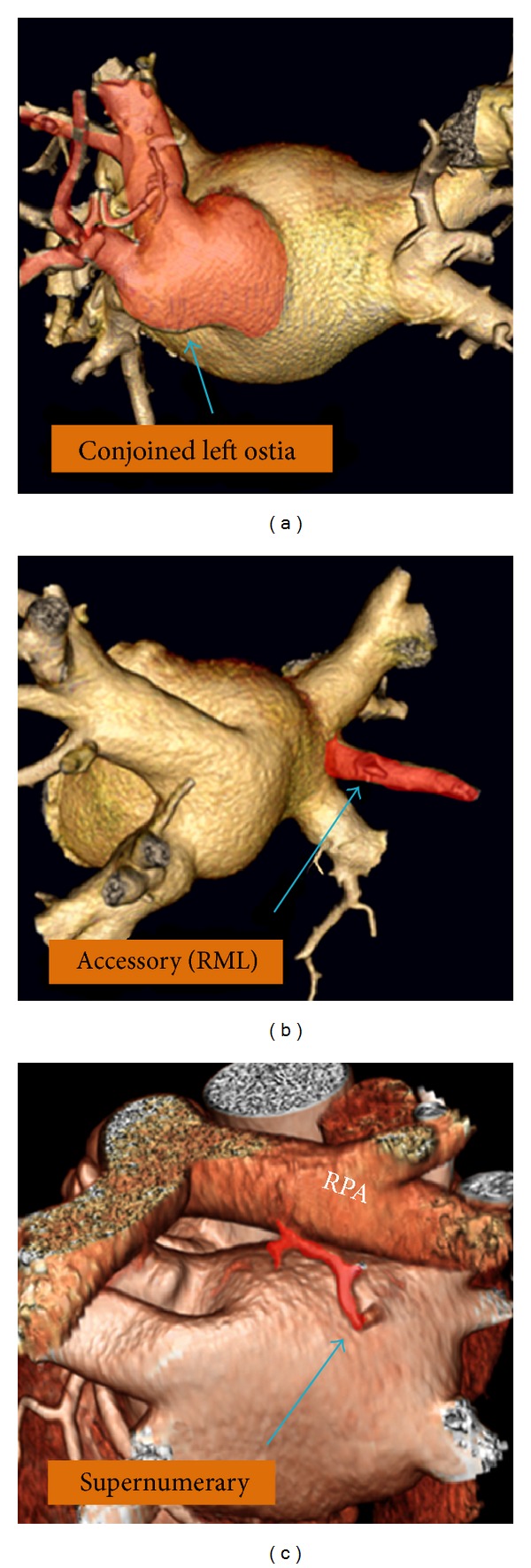
Anatomical variations of the pulmonary veins. Accessory right middle lobe (RML) and conjoined ostia are common and are seen in one fourth of studies. Accessory branches on the left side are not common. Supernumerary branch is usually a small branch found in 5–10% of chest studies. It usually drains the posterior segment of the right upper lobe and consistently travels behind the right main bronchus before entering the left atrium. Rarely, supernumerary branch arises from the superior segment of the right lower lobe and directly connects to the posteroinferior margin of the left atrium. Note close relationship of the right pulmonary artery (RPA) and the superior wall of the left atrium.

**Figure 5:**
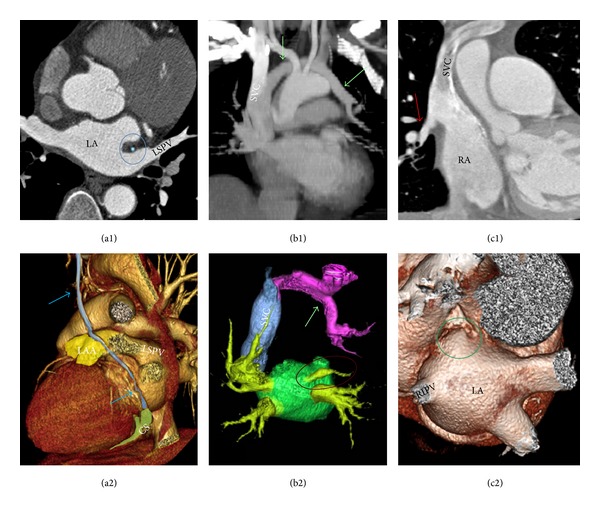
CT slices (top) and view of volume rendered CT angiographies (bottom) are shown. (a1) and (a2) demonstrate a patent vein of Marshall (blue arrow and within the blue ring) passing between the left atrial appendage (LAA) and the left superior pulmonary vein (LSPV) before joining the coronary sinus (CS). (b1) and (b2) are partial anomalous pulmonary venous (green arrows) return draining part of the left upper lung into the superior vena cava (SVC). Note that the left superior pulmonary vein exists (within red circle) but appears diminutive compared to the other veins. (c1) and (c2) show partial anomalous pulmonary venous (red arrow) return draining part of the right upper lung into the SVC. In this case, the right superior pulmonary vein is absent (green circle). RIPV = right inferior pulmonary vein. (b2) and (c2) are flipped for better orientation.

Normal PVs anatomy consists of two right-sided and two left-sided PVs with separate ostia (Figures 2 and 3). However, in anatomical studies with multidetector CT (MDCT) it has been demonstrated that the anatomy of the LA and PVs is commonly variable [23]. The PV ostia are ellipsoid with a longer superior-inferior dimension. The right superior PV is located close to the superior vena cava and the right inferior PV possesses a projection horizontally. The left superior PV is close to the LAA and the left inferior PV courses near the descending aorta. The veins are larger in AF versus non-AF patients, men versus women, and persistent versus paroxysmal patterns. The PV trunk is defined as the distance from the ostium to the first-order branch. The superior PV ostia are larger (19-20 mm) than the inferior PV ostia (16-17 mm) [24]. The superior PVs tend to have a longer trunk (21.6 ± 7.5 mm) than the inferior PVs (14.0 ± 6.2 mm) [24]. It is important to measure the ostial diameters of each vein and the length to the first-order branch. These diameters influence the selection of the circular catheter size used. Common anomalies include a conjoined (common) left or right pulmonary vein in 25% of individuals [24]. A conjoined PV is seen more frequently on the left than the right side [25]. Supernumerary veins are also frequent. The most common is a separate right middle PV, which drains the middle lobe of the lung [26] (Figure 4). One or two middle lobe vein ostia can be seen in 26% of patients [25]. The ostial diameter of the right middle PV is smaller than that of the other veins (mean, 9.9 ± 1.9 mm). In some patients, there is a supernumerary PV that shows an aberrant insertion, with a perpendicular position in relation to the LA posterior wall. The supernumerary branch usually drains the upper lobe of the right lung and characteristically passes behind the bronchus intermedius. The absence of one PV requires careful examination of the whole intrathoracic venous system since it may be associated with partial anomalous venous return (Figure 5). The caliber of the PVs gradually increases as they approach the LA. However, the caliber of the left inferior PV may decrease as it enters the LA.

**Figure 6:**
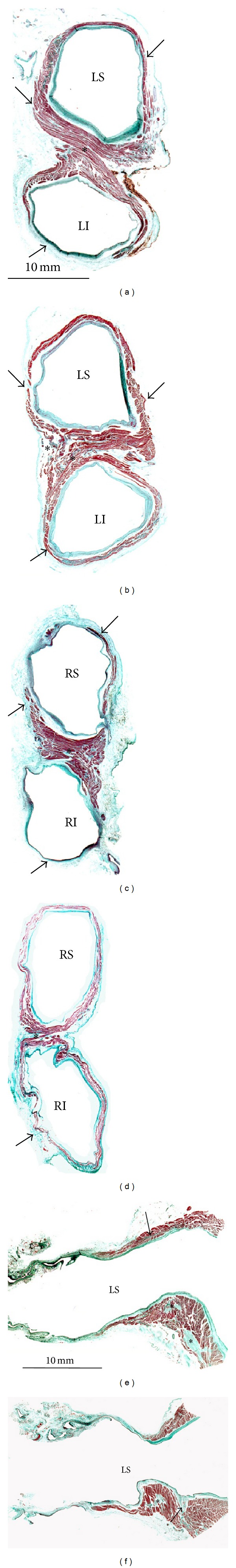
Cross-histological sections of the left (a), (b) and right (c), (d) pulmonary veins stained with Masson's trichrome showing variations in circumferential thickness of the myocardial sleeves (arrows). Note the interpulmonary myocardial connections (∗) between the superior and inferior veins and that the inferior veins tended to have less myocardial coverage than the superior veins. Longitudinal histological sections (e), (f) with Masson's trichrome stain show the thicker atrial wall becoming thinner at the entrance of the left superior pulmonary vein to form the muscular sleeve, which tapers toward the lung. Note the presence of gaps of connective tissue bridges between the myocardial fibers (arrows) and small areas of myocardial degeneration with fibrous replacement (∗). LI = left inferior pulmonary vein, LS = left superior pulmonary vein, RI = right inferior pulmonary vein, and RS = right superior pulmonary vein.

The presence of atrial myocardial tissue extending over the wall of the PVs has been confirmed both macroscopically and histologically by many investigators [27–31]. In most PVs (96%) the smooth muscle of the venous wall overlaps with a layer of myocardial bundles extending over the inner layer but is separated from it by a thin plane of fibrofatty tissue (Figures 2 and 6). This intermediate myocardial layer, between the adventitia and the venous media, is the myocardial continuity from the left atrial wall (so-called myocardial sleeves) in a fine matrix composed of collagen, elastic fibers, and blood vessels [27, 28]. Therefore, as seen in longitudinal sections, the extension of the left atrial musculature lies externally to the venous wall and within the epicardium/adventitia. In our histological study on 65 veins [27], the thickness of the smooth muscle ranged from 0.05 to 1 mm at the venoatrial junction, diminishing to 0.03–0.5 mm at a distance of 10 mm from the junction. The lengths of the sleeves varied from vein to vein, and the distal margins were irregular in most of the specimens, especially those of the inferior veins which tended to have less myocardial coverage than the superior veins. In keeping with previous anatomic studies, the sleeves in the superior right and left PVs were longer than those observed in the inferior veins. The sleeves were thickest at the venoatrial junction (1.88 ± 0.45 mm, range 1.2–2.8 mm) and then tapered toward the lung hila, but the decrease in thickness was not uniform circumferentially. When superior and inferior PVs were compared, the sleeves were thickest in the left superior veins. The anatomic orientation of the myocardial fibers making up the sleeves is highly variable (Figure 2(d)). Although the sleeves are mainly composed of circularly orientated bundles, oblique and longitudinally oriented fibers are also common [27, 30]. These architectural observations are consistent with mapping studies of the PVs in the living human heart, demonstrating conduction patterns that evidence conduction in a longitudinal and transverse direction [32]. At the level of the venoatrial junction, we found in our specimens myocardial fibers crossing the isthmus or carina between the superior and inferior venous orifices in both right and left PVs (Figures 6(a)–6(d)). These crossing fibers were found in 41% of the hearts at the level of the left PVs and in the right PVs in 25% of hearts [28]. The carina appears particularly important in the development of AF and must be considered in developing the optimal ablation strategy in many patients. Most triggers for initiating AF appear to originate from PV carina [33] which may imply isolation of contiguous vessels from a single or circumscribed region to achieve complete PV electrical disconnection.

Several studies have been designed to investigate and to compare the pathology of the PVs in patients with and without AF [29, 31]. Although patients with AF have a higher number of pathological alterations, anatomical findings are similar. Gaps of myocardial tissue of irregular morphology or discontinuous myocardium, small areas of myocardial degeneration with fibrous replacement, and presence of hypertrophic myocytes were in both groups. The presence of collagenous septa between myocardial fibers may result in progressive electrical uncoupling of the side-to-side connections between groups of parallel atrial fibers (Figures 6(e) and 6(f)). These findings are important, since they may be the basis for a nonuniform anisotropic conduction of a wavefront at a given area and for the development of reentry within smaller regions [34]. Some authors [35] have identified sinus node-like cells (P cells) within human PVs that were associated with AF. These myocardial cells were identified under light microscopy by their pale cytoplasm and their positive response to periodic acid-Schiff (PAS) staining in 4 of the 5 autopsy subjects. Whether these PAS-positive cardiomyocytes have pacemaker currents or are a potential source of automaticity remains unknown. In our series of structurally normal human hearts, we did not observe node-like cells or discrete tract of specialized myocytes.

## 5. Gross Anatomy of the Left Lateral Ridge

**Figure 7:**
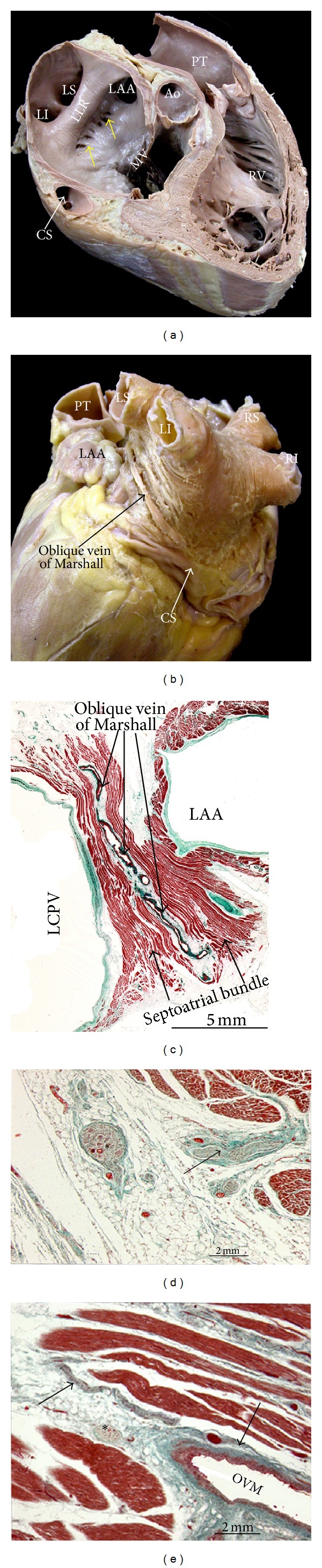
(a) Longitudinal section through the roof of the left atrium showing the endocardial view of the left atrium and right ventricle. Note the prominent left lateral ridge between the ostium of the left atrial appendage and the orifice of the left superior pulmonary vein. In this specimen, extra pectinate muscle trabeculations are extending inferiorly from the appendage to the vestibule of the mitral valve (arrows). (b) Posterolateral view of the left atrium and left ventricle to show the myofiber arrangement in the subepicardium of the left lateral ridge, left pulmonary veins, and the trajectory of the oblique vein of Marshall. Note that the vein of Marshall is in direct contact with the myocardium of the ridge. (c), (d), and (e) Cross-histological sections of the left lateral wall of the left atrium stained with Masson's trichrome. (c) shows the oblique vein of Marshall in direct contact with the myocardium of the septoatrial bundle. (d) and (e) show the epicardial ganglion (∗) and autonomic nerve bundles (arrows) in the vicinity of the oblique vein of Marshall. LAA = left atrial appendage, PT = pulmonary trunk, Ao = aorta, MV = mitral valve, RV = right ventricle, CS = coronary sinus, LI = left inferior pulmonary vein, LS = left superior pulmonary vein, LLR = left lateral ridge, RI = right inferior pulmonary vein, RS = right superior pulmonary vein, OVM = oblique vein of Marshall, and LCPV = left common pulmonary vein.

**Figure 8:**
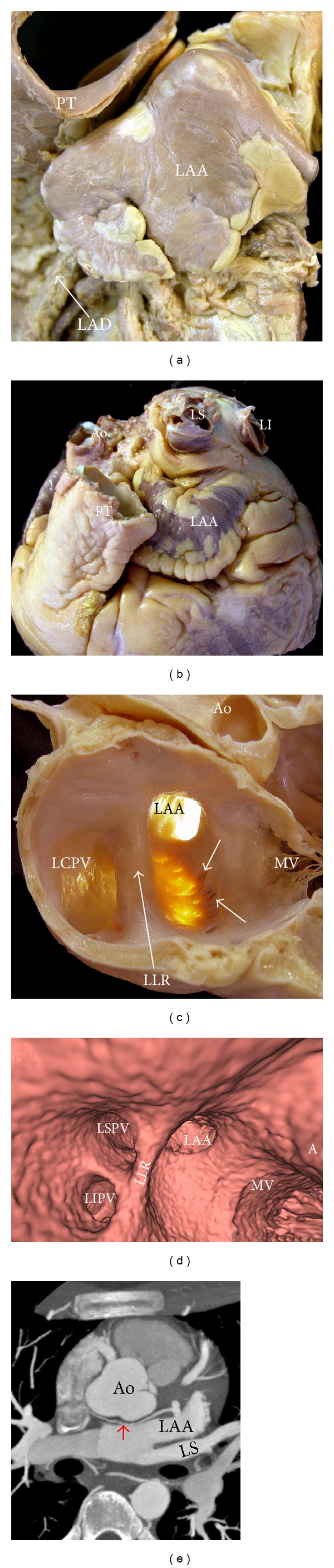
(a), (b) Images demonstrating significant interindividual variation in left atrial appendage (LAA) morphology (A: chicken wing; B: windsock). The tip of the LAA can be in a variety of positions, lying over the pulmonary trunk (PT) and the left anterior descending (LAD) coronary artery (a) or even directed medially towards the back of the aorta (b). (c) Endocardial visualization of the left posterolateral wall showing a prominent left lateral ridge (LLR) and extra pectinate muscle trabeculations (arrows) extending inferiorly from the appendage to the vestibule of the mitral valve. Transillumination in this specimen shows the atrial wall becoming exceptionally thin. (d) Endoscopic CT angiography view of the left posterolateral wall shows the relationship of the left superior pulmonary vein (LSPV) and the ostium of the LAA. In this example, both are located at the same level. The LLR is relatively thin. (e) Axial CT view at the left of the LAA shows a single lobe LAA and a thin LLR between the LAA and the left superior pulmonary vein (LS). The red arrow shows the left sinoatrial node artery arising from the left circumflex artery. Ao = aorta, LI = left inferior pulmonary vein, LS = left superior pulmonary vein, MV = mitral valve, and LCPV = left common pulmonary vein.

The left lateral ridge (LLR) between the orifices of the left PVs and the mouth of the LAA is the most relevant structural prominence of the endocardial LA [36] (Figures 1, 7, and 8). The shape and size of this ridge are of relevance during catheter ablation of AF when encircling the orifices of the left PVs or during ablation of extrapulmonary vein triggers arising around or inside the LAA. Anatomic information of this structure may be useful in order to perform ablation techniques more efficiently and safely, and it can be obtained with current MDCT and MRI reconstructions of the inner aspect of LA [37–39]. A three-dimensional MRI study showed that the ridge was narrowest between the left superior PV and the LAA in 84% of patients. In this study, the mean distance between the left superior PV and the LAA and between the left inferior PV and the LAA was found to be 3.8 ± 1.1 mm and 5.8 ± 2.0 mm, respectively. The ridge was narrower than 5 mm in the majority of patients, thus determining the possibility of obtaining stable catheter position in this region [37]. Our anatomical study demonstrated that the LLR has a narrower width and thicker myocardium superiorly rather than inferiorly [36]. The spatial relationship between the orifices of the LAA and the left superior PV (LSPV) is also of relevance. Anatomical studies using MDCT [39, 40] have shown that in most patients (58–64%) the LAA and LSPV orifices are at the same level (Figure 8(d)). The LAA orifice is shown to be located superiorly to the LSPV ostium in 22–30% of cases and inferiorly to it in 12-13% [39].

Within the LLR, the oblique vein of Marshall runs, together with abundant autonomic nerve bundles [36] and a small atrial artery, which in some cases is the sinoatrial node artery. The oblique vein of Marshall, a remnant of the left superior vena cava, descends along the lateral and inferior walls of the LA, between the LAA and the left PVs (Figure 7(b)). It joins the cardiac vein system at the junction of the great cardiac vein and the coronary sinus, approximately 3 cm away from the coronary sinus ostium [41, 42]. The vein is short (2-3 cm), and its superior part can be obliterated by fibrosis. The vein or ligament of Marshall is located in the epicardial aspect of the LLR in close proximity to the endocardial surface, at a distance of 3 mm at the superior level of the LLR in 73% of specimens, and has muscular connections to the left PVs [36]. These connections might provide a substrate for focal triggers initiating AF. By cannulating the vein, Hwang et al. [43] were able to record electrical activity in patients with focal AF arising from the vein or ligament of Marshall.

## 6. Left Atrial Isthmus and Atrial Fibrillation

**Figure 9:**
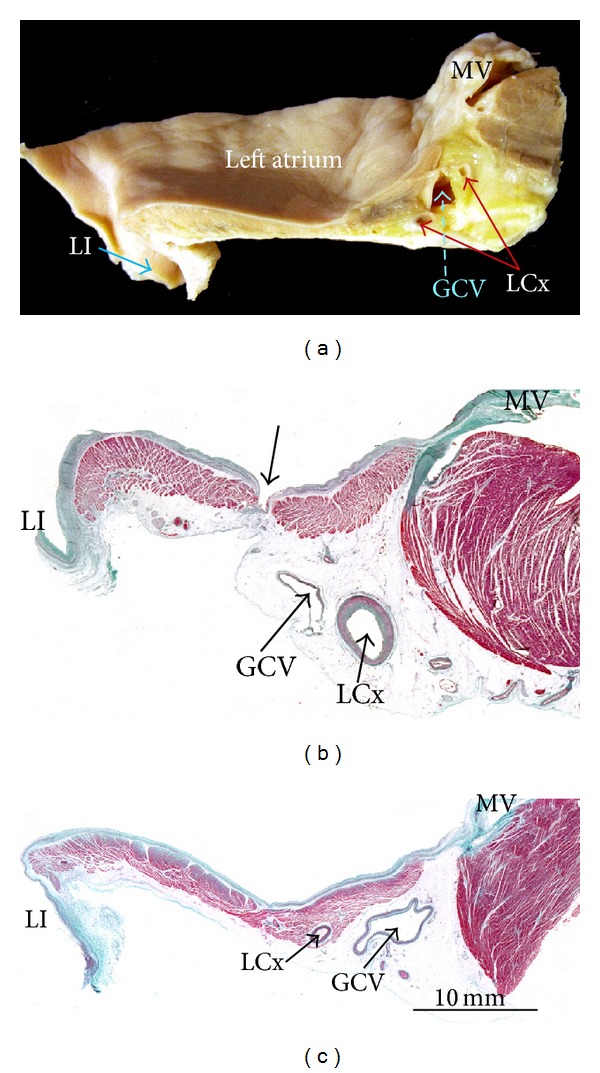
Longitudinal sections at the mitral isthmus to illustrate its anatomic relations with the great cardiac vein/coronary sinus and left circumflex artery. (a) This cut shows in profile the mitral isthmus between the mitral annulus and the orifice of the left inferior pulmonary vein. (b) and (c) These corresponding histological sections stained with Masson's trichrome show the irregular length and thickness of the atrial wall and the relationship to the great cardiac vein/coronary sinus. Note in (b) the space between the pectinate muscles where the left posterior atrial wall becomes thinner (arrow). LI = left inferior pulmonary vein, MV = mitral valve, LCx = left circumflex artery, and GCV = great cardiac vein.

**Figure 10:**
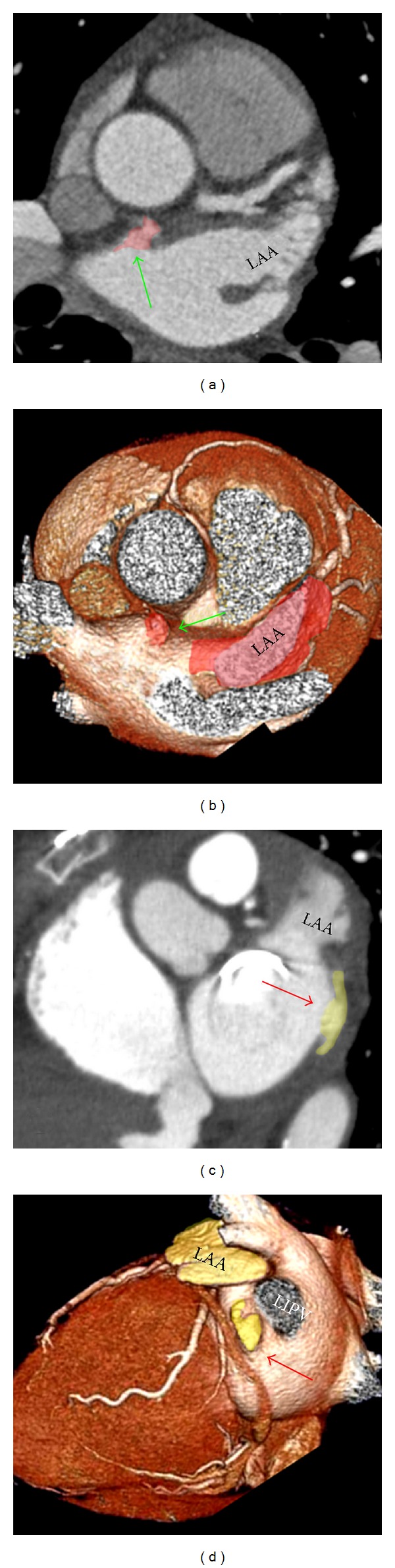
Small accessory appendages are common findings in the left atrial wall presenting as small outpouchings with irregular margin in the left atrial wall arising from the anterior wall (green arrow) and the mitral isthmus (red arrows). The left atrial isthmus is the area between the orifice of the left inferior pulmonary vein (LIPV) and the posteroinferior margin of mitral annulus.

Linear ablation connecting the inferior margin of the ostium of the left inferior PV and the mitral annulus, particularly when complete linear block is achieved, appears to increase the success rate of catheter ablation in patients with persistent or long-standing/permanent atrial fibrillation and prevent macroreentry around the mitral annulus or the left PVs [44]. Although this posteroinferior wall of the LA between the orifice of the left inferior PV and the mitral annulus cannot be considered an anatomic entity, it is being named by electrophysiologists as the left atrial isthmus or mitral isthmus (Figure 9). The creation of mitral isthmus lesions by catheter ablation is technically challenging and may be associated with significant complications. Factors that make obtaining a complete, transmural, and permanent ablation line across the mitral isthmus difficult may be electrical as well as anatomical because of the variable and complex endocardial geometry of this region. Other factors include the unpredictable content of atrial myocardium and the cooling effect of the circumflex artery at different locations of this atrial territory. In a postmortem study of 20 hearts, Becker [45] showed marked variability in the dimensions of the mitral isthmus with considerable differences in thickness of the left atrial myocardium at various levels and among different hearts. In a more recent histological examination revealed that the thickest atrial wall was midway between the mitral annulus and the left inferior PV with tapering at either end of the isthmus [46], Wittkampf et al. [46] have suggested that the muscle sleeve around the coronary sinus and the close anatomic proximity of the circumflex artery are the two major anatomic determinants for the creation of mitral isthmus conduction block. Also the presence of small crevices or pouches in 28% of the hearts close to the base of the LAA is relevant and continued with the vestibule of the mitral valve forming extra-appendicular muscular bundles that may entrap the tip of the ablation catheter, increasing the risk of isthmus perforation [36] (Figures 8, 9, and 10).

Additional linear ablation lesions are created to improve the outcomes of PV isolation during AF ablation. In their study Cho et al. [47] using multislice computed tomography analyzed the anatomical characteristics of three different endocardial lines in the left atrium anteromedial (AM), anterolateral (AL), and posterolateral (PL) from right superior, left superior, and left inferior PV, respectively. In their data, Cho et al. [47] showed that the PL lines were shortest among the three endocardial lines but closest to left circumflex artery and great cardiac vein. Myocardium was thickest at the AL line, and sinus nodal arteries were frequently found on the AM and AL lines. In addition, ridge, cord-like structure or diverticulum was found most frequently at the AM line.

## 7. Left Atrial Appendage

The LAA appears to be responsible for triggering AF in 27% of patients presenting for repeat procedures of catheter ablation [48]. The LAA is characteristically a small finger-like extension of the LA with a multilobulated appearance in 80% of hearts [49] (Figure 8). The tip of the LAA can be in a variety of positions, lying over the pulmonary trunk and the left anterior descending coronary artery, pointing posteriorly, or even directed medially towards the back of the aorta [50]. A quantitative study of the normal LAA in 500 autopsy hearts showed that the mean length, width, and size of the appendage increased with age upto 20 years [49]. In a recent study, Di Biase et al. [51] analyzed gross morphology of the LAA in patients with AF using CT and MRI. The LAA morphologies were classified as cactus (30%), chicken wing (48%), windsock (19%), and cauliflower (3%). The study showed that patients with chicken wing LAA morphology were less likely to have an embolic event.

In autopsy specimens and imaging studies, the LAA ostium is usually elliptical or round and in elliptical-shaped variant and its long axis is obliquely orientated relative to the mitral annulus [52, 53]. The LAA interior surface is lined by complicated network of fine pectinate muscles. The pectinate muscles have been thicker than 1 mm in most of our autopsy hearts (95–97%). Pectinate muscles <1 mm in size have been noted only in the specimens from the first or last decade of life. Anselmino et al. [54] correlated the LAA morphology and the amount of pectinate trabeculations using MDCT. They observed mild trabeculations in LAAs with a chicken wing morphology, moderate trabeculations in cases with a cactus morphology, and extensive trabeculations in LAAs with a cauliflower morphology. Although cauliflower morphology with extensive trabeculations was the uncommon type in their series (5.2%), they were more likely to have a silent cerebral ischaemia.

In some specimens (28%), muscular trabeculations can be found extending inferiorly from the appendage to the vestibule of the mitral valve [36]. These extra-appendicular myocardial bands correspond to the small set of posterior pectinate muscles originating from the myocardial bundles to embrace the LAA. In those hearts with extra-appendicular posterior pectinate muscles (Figures 7(a), 8(c), and 9(b)), the areas between the muscular trabeculae and the atrial wall become exceptionally thin (0.5 ± 0.2 mm), increasing the risk of cardiac perforation during ablation.

## 8. Important Structures in the Neighborhood of the Left Atrium

### 8.1. Relationship between the Esophagus and Left Atrium

**Figure 11:**
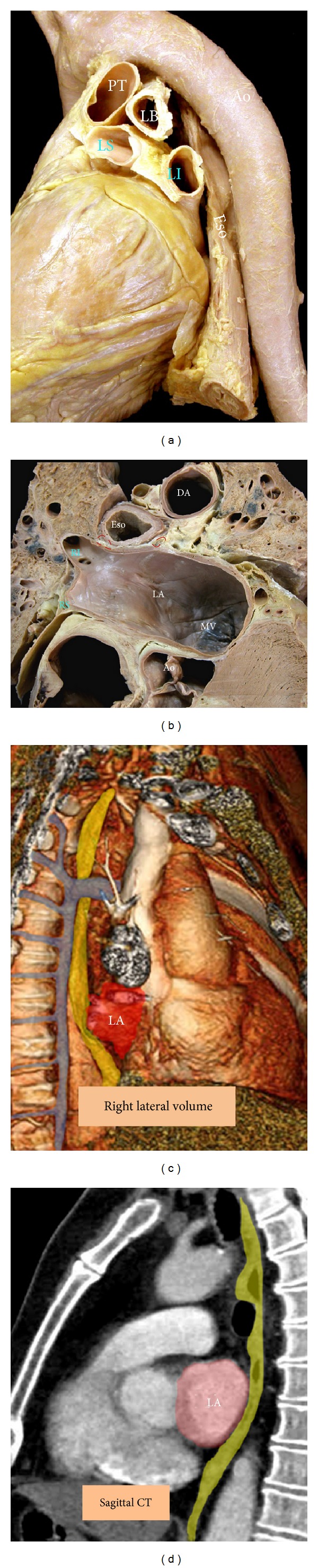
(a) Specimen viewed from a left posterolateral perspective to show the course of the esophagus (Eso) and descending aorta (DA) relative to the left atrium (LA). (b) An overview of a transthoracic section through a cadaver showing the locations of the esophagus, the descending aorta, and the vagus nerves (red dashed circles). Note the minimal distance between the endocardial surfaces of the right pulmonary vein. (c) Right lateral volume rendered CT angiography and (d) sagittal CT of chest show the relationship of the esophagus (yellow) to the left atrium (red). The azygos is shown in blue. LI = left inferior pulmonary vein, LS = left superior pulmonary vein, MV = mitral valve, PT = pulmonary trunk, Ao = aortic valve, LB = left bronchus, RI = right inferior pulmonary vein, and RS = right superior pulmonary vein.

Due to the close proximity of the esophagus to the posterior wall of the LA, ablation procedures involving this region of the LA may cause esophageal damage and result in the formation of an atrial esophageal fistula [55] (Figure 11). In an anatomic study, we examined the course of the esophagus in 15 cadavers [11]. It is important to recognize that the esophagus follows a variable course along the posterior aspect of the LA. It was <5 mm from the endocardium in 40% of specimens. In 40% of cases, it passed along the middle portion of the posterior LA wall. In 20% of specimens, it descended close to the right venoatrial junction; in the remaining cases, it had a leftward course close to the left venoatrial junction [11]. Behind the posterior left atrial wall is a layer of fibrous pericardium and fibrofatty tissue of irregular thickness that contains esophageal arteries and the vagus nerve plexus. These anatomic structures may be affected by ablative procedures. MDCT is valuable tools for showing the relationship of the atrial wall and the oesophagus and the descending aorta prior to the ablation procedure [56] (Figure 11). However, peristalsis and dynamic movement of the esophagus during the procedure can result in discordance between the preprocedure and intraprocedure anatomy.

### 8.2. Periesophageal Vagal Nerves

Thermal injury may involve the periesophageal vagal nerves, resulting in acute pyloric spasm and gastric hypomotility [57]. The vagus nerves pass behind the root of the lungs and form right and left posterior pulmonary plexuses (Figure 11). From the caudal part of the left pulmonary plexus, two branches descend on the anterior surface of the esophagus joining with a branch from the right pulmonary plexus to form the anterior esophageal plexus. The posterior and anterior esophageal plexuses enter the abdomen through the esophageal diaphragmatic opening, reuniting to become the posterior and anterior vagal trunks that innervate the stomach and pyloric canal and the digestive tract as far as the proximal part of the colon. An anatomical study revealed a mean distance between the bundles of the anterior esophageal plexus and posterior LA endocardium or venoatrial junctions of 4.1 ± 1.4 mm (range: 2.5–6.5 mm) [58].

### 8.3. The Phrenic Nerves

**Figure 12:**
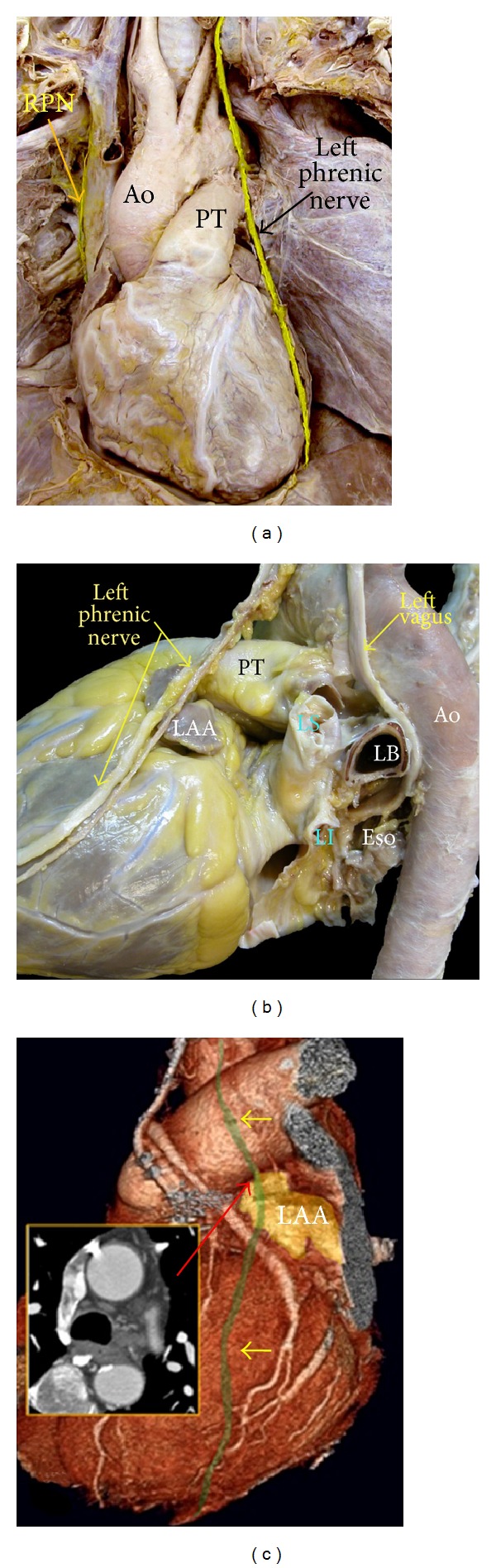
(a) This dissection of a cadaver viewed from the front shows the course of the right and left phrenic nerves. (b) Dissection of the left phrenic nerve, which descends onto the fibrous pericardium anterior and lateral to the aortic arch, alongside the distal part of pulmonary trunk (PT), left atrial appendage (LAA), and the lateral wall of the left ventricle. (c) Left lateral volume rendered CT image of the heart shows the anatomic course of left phrenic neurovascular bundle (shown in green and demarcated by yellow arrows) in relation to the LAA. Corresponding two-dimensional axial CT at the superior margin of the LAA (red arrow) is also shown (inlay images). Neurovascular bundle is usually seen as a single trunk anterior or over the body of LAA. In 23% of individuals, the nerve moves over the neck of LAA. LS = left superior pulmonary vein, Ao = aorta, Eso = esophagus, LB = left bronchus, RPN = right phrenic nerve, and LI = left inferior pulmonary vein.

The phrenic nerves lie along the lateral mediastinum and run from the thoracic inlet to the diaphragm (Figure 12). Phrenic nerve injury results from direct thermal injury, usually to the right phrenic nerve, which is located near the right superior PV and the superior vena cava [59, 60]. Tracing the course of the RPN revealed its close proximity of the SVC (minimum 0.3 ± 0.5 mm) and the RSPV (minimum 2.1 ± 0.4 mm). The anterior wall of the RSPV was <2 mm from the RPN in 32% of autopsy specimens [59]. Less frequently, ablation within the LAA can result in left phrenic nerve damage. The endocardium of the roof of the LAA was <4 mm from the LPN in 2 (9%) specimens. The nerve passed <2.5 mm from the epicardium of the apex of the LAA in 7 (31%) specimens [61]. MDCT coronary angiography can demonstrate the left phrenic neurovascular bundle as it passes over the LV pericardium in 74% of the studies [62] (Figure 12(c)). Demonstrating the right phrenic neurovascular bundle with CT is possible but in a short distance near the right pulmonary hilum.

### 8.4. Intrinsic Left Atrial Innervation

The intrinsic cardiac nervous system (ICNS) is a crucial regulator of heart rate, atrial and ventricular refractoriness, contractility, and coronary blood flow. Morphologically, the ICNS forms a neural ganglionated plexus that may be subdivided into epicardial and myocardial subplexuses. Several studies indicate that the ICNS is a complex of distinct subplexuses and that the cardiac ganglia are mainly distributed on (1) the superior surface of the right atrium, (2) the superior surface of the left atrium, (3) the posterior surface of the right atrium, (4) the posterior medial surface of the left atrium (the latter two fuse medially where they extend anteriorly into the interatrial septum), and (5) the inferior and lateral aspect of the posterior left atrium and left PVs [63, 64]. Postganglionic neurons distributed within discrete fat pads on the atrial surface mediate certain aspects of cardiac function. For example, ganglion cells in a fat pad adjacent to the epicardium of the right PVs selectively mediate negative chronotropic effects [65], whereas ganglion cells in a fat pad at the junction between the inferior right atrium and the inferior the caval vein mediate AV conduction slowing [66].

## 9. Conclusions

Catheter ablation has been shown to be an increasingly important therapeutic option for patients with paroxysmal, persistent, and chronic AF. Ablation techniques have evolved from rather limited initial approaches to quite extensive atrial interventions. The LA has a distinctive atrial appendage and an atrial body that comprises component parts that blend into one another. The patterns of general myocardial arrangement in the left atrial wall and the presence of interatrial muscle bundles may provide some anatomic background to atrial and interatrial conduction. Understanding the structure of the component parts and their relationship to one another and to other cardiac structures is relevant to interventional procedures inside and outside of the LA.
