# Corrigendum to “Left Atrial Anatomy Relevant to Catheter Ablation”

**DOI:** 10.1155/2020/3490543

**Published:** 2020-08-11

**Authors:** Damián Sánchez-Quintana, José Ramón López-Mínguez, Yolanda Macías, José Angel Cabrera, Farhood Saremi

**Affiliations:** ^1^Department of Anatomy and Cell Biology, Faculty of Medicine, University of Extremadura, Badajoz 06006, Spain; ^2^Department of Cardiology, Hospital Infanta Cristina, Badajoz 06080, Spain; ^3^Department of Cardiology, Hospital Quirón, European University of Madrid, Madrid 28223, Spain; ^4^Department of Radiology, University of Southern California, Los Angeles, CA 90089, USA

The article titled “Left Atrial Anatomy Relevant to Catheter Ablation” [1] was found to contain material, without citation, from the following published articles:

- José Angel Cabrera, Jerónimo Farré, Siew Yen Ho, and Damián Sánchez-Quintana, “Anatomy of the Left Atrium Relevant to Atrial Fibrillation Ablation,” Catheter Ablation of Atrial Fibrillation, 2009 (https://doi.org/10.1002/9781444300185.ch1) [2].
- Damián Sánchez-Quintana, Gonzalo Pizarro, José Ramón López-Mínguez, Siew Yen Ho, and José Angel Cabrera, “Standardized Review of Atrial Anatomy for Cardiac Electrophysiologists,” Journal of Cardiovascular Translational Research (2013) 6: 124 (https://doi.org/10.1007/s12265-013-9447-2) [3].
- Farhood Saremi and Damián Sánchez-Quintana, “Anatomy for Electrophysiologic Interventions,” Revisiting Cardiac Anatomy: A Computed-Tomography-Based Atlas and Reference, 2010 (https://doi.org/10.1002/9781444323191.ch11) [4].
- Damián Sánchez-Quintana and Margarita Murillo, “Anatomical Bases of Supraventricular Arrhythmias,” European Journal of Anatomy, 15 (1): 17–28 (2011) (http://eurjanat.com/data/pdf/eja.110006ds.pdf) [5].
- Farhood Saremi and Mona Tafti, “The Role of Computed Tomography and Magnetic Resonance Imaging in Ablation Procedures for Treatment of Atrial Fibrillation,” Seminars in Ultrasound, CT and MRI, 2009 (https://doi.org/10.1053/j.sult.2008.10.015) [6].

The article [1] includes an overlap of 3,257 words with José Angel Cabrera et al. [2], 1,536 words with Damián Sánchez-Quintana et al. [3], 1,208 words with Farhood Saremi and Damián Sánchez-Quintana [4], 923 words with Damián Sánchez-Quintana and Margarita Murillo [5], and 883 words with Farhood Saremi and Mona Tafti [6].

The authors clarified that they did not cite these articles because they did not consider it relevant to include citations to their previous work, unnecessarily lengthening the reference list.
